# Balancing Immune Protection and Immune Pathology by CD8^+^ T-Cell Responses to Influenza Infection

**DOI:** 10.3389/fimmu.2016.00025

**Published:** 2016-02-05

**Authors:** Susu Duan, Paul G. Thomas

**Affiliations:** ^1^Department of Immunology, St. Jude Children’s Research Hospital, Memphis, TN, USA

**Keywords:** CD8^+^ T cells, influenza, human, immune regulation, immunopathology, vaccination

## Abstract

Influenza A virus (IAV) is a significant human pathogen causing annual epidemics and periodic pandemics. CD8^+^ cytotoxic T lymphocyte (CTL)-mediated immunity contributes to the clearance of virus-infected cells, and CTL immunity targeting the conserved internal proteins of IAVs is a key protection mechanism when neutralizing antibodies are absent during heterosubtypic IAV infection. However, CTL infiltration into the airways, its cytotoxicity, and the effects of produced proinflammatory cytokines can cause severe lung tissue injury, thereby contributing to immunopathology. Studies have discovered complicated and exquisite stimulatory and inhibitory mechanisms that regulate CTL magnitude and effector activities during IAV infection. Here, we review the state of knowledge on the roles of IAV-specific CTLs in immune protection and immunopathology during IAV infection in animal models, highlighting the key findings of various requirements and constraints regulating the balance of immune protection and pathology involved in CTL immunity. We also discuss the evidence of cross-reactive CTL immunity as a positive correlate of cross-subtype protection during secondary IAV infection in both animal and human studies. We argue that the effects of CTL immunity on protection and immunopathology depend on multiple layers of host and viral factors, including complex host mechanisms to regulate CTL magnitude and effector activity, the pathogenic nature of the IAV, the innate response milieu, and the host historical immune context of influenza infection. Future efforts are needed to further understand these key host and viral factors, especially to differentiate those that constrain optimally effective CTL antiviral immunity from those necessary to restrain CTL-mediated non-specific immunopathology in the various contexts of IAV infection, in order to develop better vaccination and therapeutic strategies for modifying protective CTL immunity.

## Introduction

Influenza A virus (IAV) causes acute respiratory tract infection and is a significant human pathogen causing annual epidemics and periodic pandemics (1). Annual influenza epidemics are caused by circulating “seasonal” influenza viruses, which currently include H1N1 and H3N2 subtype IAVs and influenza B viruses. The RNA genome of IAV has high mutation rates due to the high error rate of its RNA polymerase, allowing the viruses to quickly evolve under selection pressures to develop antigenically drifted strains. Occasional influenza pandemics are caused by the introduction of novel antigenically shifted strains to human populations. These strains, including 2009 pandemic H1N1 IAV, often result from the reassortment of different zoonotic IAVs (2). Reassortment also can generate antigenically distinct new subtypes that pose a pandemic threat to the human population, such as the recent outbreak of human infections of zoonotic H7N9 IAV in China (3). Due to their ability to constantly generate new strains through mutation and reassortment, IAVs pose continuing threats to human populations.

Upon encountering IAVs, the complex host immune system senses the invasion and then mounts innate and adaptive immune responses intended to clear the virus. Multiple arms of host immunity are used for protection against influenza infection. The innate responses initiate inflammation, limit virus replication, and provide signals to activate adaptive immunity (4). The ultimate control of virus replication and clearance of virus-infected cells rely on the virus-specific adaptive immune responses, including antibodies and CD4^+^ and CD8^+^ T cells (5). IAV-specific antibodies bind and neutralize viral proteins (neutralizing antibodies) or mediate other virus clearance activities (non-neutralizing antibodies) in cooperation with immune cells, including CD8^+^ T cells and lung-resident phagocytes (6–10). CD4^+^ T cells help B cells for antibody production and CD8^+^ T cells for activation and proliferation. It has been long acknowledged that CD8^+^ cytotoxic T lymphocyte (CTL)-mediated immunity contributes to virus clearance through cytolysis of the virus-infected target cells and production of cytokines that further enhance antiviral inflammation (11). However, there is accumulating evidence that the IAV-specific CD8^+^ T cells and their effector mediators also contribute to immunopathology during IAV infection. A variety of mechanisms have been uncovered to regulate the magnitude and effector activities of IAV-specific CTL responses for effective virus clearance while limiting inflammation during influenza infection.

Here, we first review the historical advances in understanding protective and immunopathogenic roles of IAV-specific CTL responses using either IAV infection or non-viral infection models. Next, we focus on the current state of knowledge for how various regulatory mechanisms control immune protection and pathology by CTL responses during influenza infection. This is followed by a discussion of clinical findings about the role of CTL responses in human IAV infections, highlighting the evidence that emerged after the 2009 H1N1 pandemic and recent outbreaks of human infection by H7N9 IAVs. Finally, we summarize our current understanding of the multiple layers of host and viral factors mediating the outcome of antiviral CTL responses and suggest future key research directions.

## IAV Infection Induces Antiviral CD8^+^ T-Cell Responses

The mammalian airways have very large mucosal surfaces for gas exchange. IAV infection begins with virus invasion and replication in the upper airway epithelium, and from there, it can spread further into the lower airways and lung, causing severe infection. Meanwhile, the lung epithelial and endothelial cells and other airway resident innate cells, including several dendritic cell (DC) subsets, alveolar macrophages, and innate lymphoid cells, sense the viruses and initiate innate inflammation, acting as the first line of defense (4). Innate and epithelial cells use specific pattern recognition receptors (PRRs) to recognize various viral products containing pathogen-associated molecular patterns (PAMPs), including endosome-bound TLR3 (dsRNA), TLR7/8 (ssRNA), cytosolic RIG-I (ssRNA), and NLRP3 (viral ssRNA) (12). Innate sensing of viruses initiates production and/or release of various inflammatory cytokines, including type I interferon (IFN), IL-1β, IL-6, and TNF-α. These cytokines induce multiple mechanisms in the infected cells to limit virus replication and also stimulate DC activation for effective antigen acquisition and presentation to initiate IAV-specific adaptive responses.

At least three subsets of lung-resident DCs have been found to play a role in responding to IAV infection in mice (13, 14). Lung plasmacytoid DCs (pDCs) are classic potent producers of type I IFN after detecting virus (15) but were initially thought to be dispensable for antiviral T-cell immunity (16). However, recent studies found that pDCs also migrate into lung-draining lymph nodes (LNs) to play a different role in certain IAV infection contexts (17, 18) that will be discussed later in this review. Two subsets of lung DCs, CD103^+^ and CD11b^+^ DCs, are migratory DCs (19, 20) that bridge innate and adaptive immunity during influenza infection. After being activated by virus infection and innate cytokines, they migrate out of the inflamed lung into the lung-draining LNs in a CCR7-mediated way (21), carrying the acquired viral antigen; in the LNs, these migratory DCs either directly present antigens to naive T cells or transfer the antigen to other specific LN DC populations for further presentation to naive T cells (19). Naive T cells in the LNs recognize the presented IAV-specific antigens using their T-cell receptors (TCRs) and receive various costimulation signals from the activated DCs, sequentially undergoing clonal selection, expansion, activation, and differentiation into IAV antigen-specific effector CD4^+^ and CD8^+^ T cells. Studies have shown that, following IAV infection, accelerated migration of DCs carrying antigens from the infected lung to LNs occurs only during the early phase of infection (the first 36 h after infection) (22, 23), and activation of the naive T cells in the LNs then occurs in an ordered, sequential fashion, in accordance with the tempo of the DC migration (23).

Influenza A virus infection drives CD4^+^ T-cell differentiation mainly into Th1 effector T cells. These “helper” cells provide help to B cells and CD8^+^ T cells in different ways via costimulatory signals and cytokines. Notably, CD4^+^ T-cell help is not essential for the primary effector CD8^+^ T-cell responses (24), although it is important for the generation of the optimal magnitude of memory CD8^+^ T-cell responses after IAV infection (25). Effector CD8^+^ T cells then migrate from the LNs into the infected and inflamed lung where the survival and proliferation of the CTLs are further shaped by interactions with various non-migratory DC populations in lung (26–28). IAV-specific effector CD8^+^ T cells are cytotoxic cells equipped to specifically recognize and kill the virus-infected cells and produce various cytokines.

Following IAV clearance, the generated virus-specific B cells and CD4^+^ and CD8^+^ T cells undergo a rapid contraction phase with only a small proportion surviving and differentiating into memory cells, which form the memory pool necessary to protect against future infections with the same or similar viruses. During this process, chemokine receptors CCR5 and CXCR3 are important in regulating the virus-specific CTL contraction and memory generation within the infected lung (29, 30). Studies have shown that residual IAV antigen depots in lung-draining LNs can persist for weeks after virus clearance; the prolonged presentation of the residual antigen does not prime new naive CD8^+^ T cells (31) but helps to maintain the large number of the memory CD8^+^ T cells in the draining LNs or in the lung airways possibly by regulating circulation patterns and activation states of the local memory CD8^+^ T cells (32–34). However, in contrast to the lung populations, this antigen is not required for maintenance and circulation of the peripheral memory CD8^+^ T cells (35). Recently, three major subsets of memory T cells are recognized based on their anatomical location, migration patterns, and additional phenotypic and effector markers: central memory T cells (Tcm), effector memory T cells (Tem), and tissue resident memory T cells (Trm). The biology of these different subsets is outside the scope of the current review.

It is worth noting that during the differentiation of naive CD8^+^ T cells into effector CTLs and then into distinct memory subsets, the cells undergo massive transcriptional programing that regulates their effector or memory potential (36). IRF4 (37, 38) and Blimp1 (39) have been found as important transcription factors that regulate CTL clonal expansion, effector differentiation, and/or effector activity during IAV infection.

In the following review, we discuss how the activation, proliferation, survival, migration, localization, and effector activity of IAV-specific CD8^+^ T cells are tightly regulated by antigen load, multiple cytokines, and costimulatory and/or coinhibitory signals provided by various cells in the LNs and in the infected lung. Appropriate control of the magnitude and effector activity of IAV-specific CD8^+^ cells is required both for an effective antigen removal and for limiting the potential immunopathology.

## IAV-Specific CD8^+^ T Cells are Cytotoxic and Cytokine/Chemokine-Producing Cells

Influenza A virus-specific CD8^+^ CTLs play a vital role in eliminating IAV-infected host cells in the lung through their two well-defined effector activities: antigen-specific cytotoxicity and cytokine/chemokine production. The CTLs target only virus-infected host cells, using their TCRs to recognize specific viral peptides (p) in complex with host major histocompatibility complex (MHC) molecules (pMHC) on the cell surface. TCR recognition and engagement of pMHC is a prerequisite for subsequent CTL cytotoxicity and cytokine production. CTL cytotoxicity is mediated in three known ways: perforin/granzyme-mediated cytolysis, apoptosis mediated by FasL/Fas, and TRAIL/TRAIL-DR signaling (40–43). CTLs contain a pore-forming protein (perforin) and cytotoxic granules comprising proapoptotic proteases called granzymes. Perforin complexes form pores between the CTL and target cell, and the granules then release granzymes into the target cells through these pores, with granzyme B one of the most abundant (40). CTLs also use two membrane-bound TNF family ligands, FasL and TRAIL, for targeted cell killing. Engagement of these molecules with their cognate receptors (Fas and TRAIL-DR) on infected cells initiates an apoptotic signaling cascade (41–43).

Influenza A virus-specific CD8^+^ CTLs are also equipped to produce a range of cytokines and chemokines. Classically, IFN-γ and TNF-α are the most prominent effector cytokines produced by CTLs, and cells producing them are often referred to as classical “Tc1” cells. IFN-γ is a potent antiviral cytokine. It can enhance the cytotoxicity of other immune cells, promote further activation of DCs, and help B cells to promote antibody isotype switching (44). TNF-α is primarily a proinflammatory cytokine, induces non-specific death of infected cells, and regulates the function of other immune cells through TNFRs (45). CTLs are not the main source of IL-2, but a small proportion of CTLs can produce IL-2 after receiving certain costimulatory signals, which in return may provide proliferation and survival signals to themselves and other CTLs (46). CTLs producing IFN-γ, TNF-α, and IL-2 are considered more potent than those producing only one or two cytokines (47). Different subsets of IAV antigen-specific CTLs differ in their ability to produce combinations of the three cytokines (48).

IL-10 is usually produced by regulatory CD4^+^ T cells (Treg) and/or helper CD4^+^ T cells. It is commonly recognized as an immune regulatory/anti-inflammatory cytokine that serves as a brake on ongoing inflammation. IL-10 was found to be produced in large quantities in IAV-infected lungs, and surprisingly, the effector CD8^+^ T cells that simultaneously produced IFN-γ contributed a large fraction of IL-10 (49). Blocking the action of IL-10 during IAV infection resulted in enhanced pulmonary inflammation and lethal injury (49). Thus, CD8^+^ T cells produce both anti-inflammatory IL-10 and antiviral IFN-γ to fine-tune the CTL activity to an effective but restrained level.

Recently, studies found small subsets of CD8^+^ effector T cells in the IAV-infected lung that could produce non-classical CTL cytokines (50–53): “Tc2” cells producing IL-4, IL-5, and IFN-γ and “Tc17” cells producing IL-17 and IFN-γ. *In vitro* polarized Tc2 and Tc17 cells are as cytotoxic as Tc1 cells, and the adoptive transfer of Tc2 or Tc17 cells into infected mice provided different levels of survival protection after otherwise lethal IAV infection (50, 52, 53). Relative to Tc1 cells, Tc2 and Tc17 cells account for a very small proportion of effector CD8^+^ T cells *in vivo*, and the extent of their effector activity during IAV infection *in vivo* needs to be further defined.

**Figure 1:**
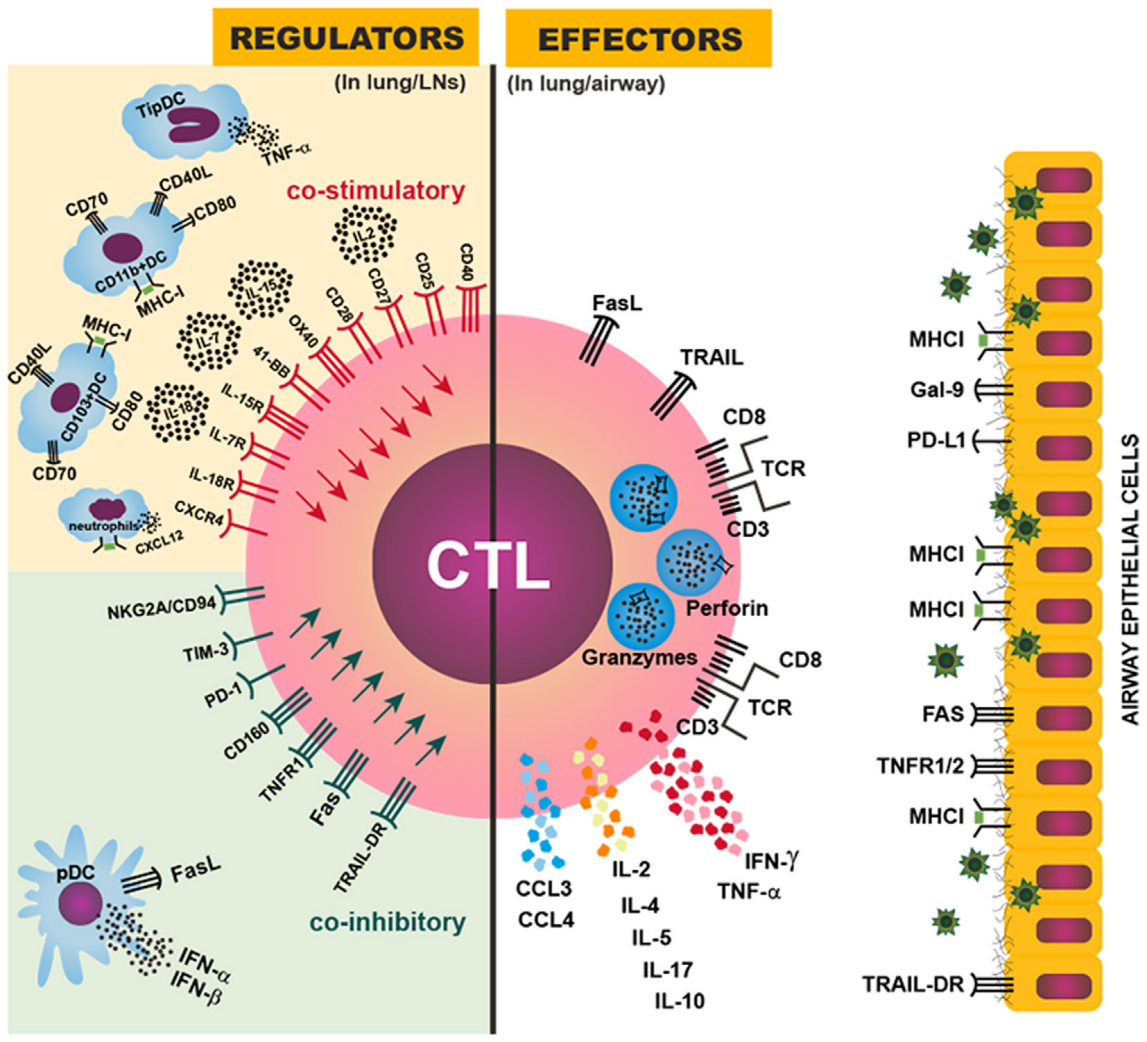
**Regulation of CTL magnitude and effector activity**. Right: CTL effector mechanisms against IAV in the infected lung or airway: the IAV-specific CTL targets IAV-infected airway epithelial cells by recognizing a viral peptide presented by MHCI molecules on the surface of infected cells; the CTL then induces cell death in the targeted cell through perforin/granzyme, FasL/Fas, and/or TRAIL/TRAIL-DR signaling; CTLs also can produce IFN-γ, TNF-α, IL-2, CCL3, CCL4, and other cytokines and chemokines to further enhance inflammation and immune activation in the infected lung. Left: various regulatory mechanisms to control the magnitude or effector activity of CTLs though costimulatory (upper) or coinhibitory (lower) signals provided in the lung-draining LNs or the infected lung. An optimal magnitude of protective CTL responses is achieved by balancing the costimulatory and coinhibitory signals, and dysregulation or imbalance among those signals can result in insufficient or exuberant CTL responses, leading to inefficient viral control or damaging immunopathology.

The two CTL effector activities (cytotoxicity and cytokine production) are precisely regulated in the infected lung by a variety of factors, including their anatomic localization and their interactions with different antigen-presenting cells with diverse pMHC density and costimulatory signals, to achieve effective target cell killing while limiting non-specific inflammation (Figure 1). These mechanisms will be discussed in detail below.

## IAV-Specific CD8^+^ T Cells are Crucial for Virus Clearance and Provide Protection during IAV Infection

The role of CTLs in clearing IAV has been demonstrated in multiple studies using adoptive transfer of IAV-specific CTLs into naive recipient mice (Table 1). In these studies, after the adoptive transfers, lung virus titers and/or the time to virus clearance were reduced, leading to accelerated recovery from non-lethal infection or survival of otherwise lethal infection (54–56). The contribution of CTLs to protective anti-IAV immunity is further corroborated by studies using β2-M-deficient mice, which are defective in MHCI complex assembly and antigen presentation and thus fail to produce functional CD8^+^ T cells (57). The β2-M-deficient mice showed a significantly delayed pulmonary virus clearance after non-lethal IAV infection and a significantly higher mortality rate after a lethal IAV infection than the control β2-M heterozygous mice (57), showing that CD8^+^ T-cell immunity is important in protection against IAV infection. However, both the β2-M-deficient mice and mice depleted of CD8^+^ T cells were able to eventually clear the virus and recover from non-lethal IAV infection (58), suggesting that the CTL response is not the sole effector of antiviral immunity during IAV infection. IAV-specific immunity consists of multiple immune mechanisms, including CTLs, antibodies, and CD4^+^ T-cell responses, which promote IAV clearance and host protection.

**Table 1:** **Overview of studies demonstrating immune protection by the CD8^+^ T-cell responses during IAV infection**.

Experimental model	IAV subtype/infection type/pathogenicity	Disease outcome after IAV infection	Measured CD8^+^ T-cell properties		Conclusion about CD8^+^ T cells	Reference
			Effector mediator	Antigen specificity		
Adoptive transfer of IAV-primed lymphocytes into naive mice	A/WSN(H1N1); lethal dose	Lower lung virus titers in recipient vs. non-recipient mice	IAV-specific cytotoxicity	Homologous IAV-primed effector lymphocytes	T cells are protective	(54)
Adoptive transfer of IAV-primed lymphocytes into naive BALB/c mice or naive nude mice	Mouse-adapted A/Port Chalmers/l/73 (H3N2, MRC-9); lethal infection	Lower lung virus titer and greater recovery in recipient vs. non-recipient mice, but no significant protection against lethal infection	IAV-specific cytotoxicity	Homologous IAV-primed effector lymphocytes	T cells are protective	(55)
Adoptive transfer of IAV-specific CD8^+^ T-cell clones into naive mice	X31(H3N2) and A/JAP/305/57(H2N2) for non-lethal infection; A/PR/8/34(HINI) for lethal infection	Lower lung virus titer after non-lethal infection; complete survival after lethal infection in recipient vs. non-recipient mice	IAV-specific cytotoxicity	Kd-restricted, NP-specific BALB/c clones T9/13 and BA4; Db-restricted, NP-specific C57BL clones B4 and B8	IAV-specific CD8^+^ T cells are protective	(56)
Wt and B2-M-deficient mice	A/Port Chalmers/1/73 (H3N2) for non-lethal infection; A/PR/8/34(H1N1) for lethal infection	Later virus clearance after non-lethal infection and greater mortality after lethal infection in B2-M-deficient vs. heterozygous mice, but virus could be cleared in the B2-M-deficient mice	IAV-specific cytotoxicity	Not described	IAV-specific CD8^+^ T cells are protective	(57)
Fas-deficient mice; chimeric mice with T lymphocytes with/without perforin deficiency into mice with/without perforin deficiency	X31(H3N2) infection	Higher lung virus titer and later virus clearance in Fas-deficient mice; perforin-deficient CD8^+^ T cells resulted in later virus clearance in wt mice but uncontrolled virus titer in Fas-deficient mice	Not described	Not described	CTLs clear IAVs via perforin- and/or Fas-dependent processes	(41)
Adoptive transfer of IAV-specific, *in vitro* polarized Tc1 or Tc2 HA-specific CD8^+^ T cells into naive mice	A/PR/8/34(H1N1) for lethal infection	Tc1 effectors but not Tc2 effectors reduced lung virus titer during early infection; Tc1 effectors conferred higher survival protection than Tc2 effectors against lethal infection	Tc1 and Tc2 were equally cytotoxic but produced different cytokines and chemokines; localized in the lung differently	Kd-restricted, HA-specific CD8^+^ T cells from a BALB/c clone 4 TCR transgenic mice; *in vitro* polarized Tc1 or Tc2 effectors	Tc1 is more protective than Tc2 effectors	(50)
Same as above, Tc1 and Tc2 mice with/without IFN-γ deficiency were compared	A/PR/8/34(H1N1) for lethal infection	IFN-γ-deficient Tc1 cells were equally effective in viral control; IFN-γ-deficient Tc2 cells were effective in viral control but showed the severest impairment of lung function	Same as described above	Same as above but with/without IFN-γ deficiency	Tc2 but not Tc1 depends on IFN-γ for its protective role	(51)
Adoptive transfer of *in vitro* polarized Tc17 and Tc1 OT-I CD8^+^ T cells into naive mice	OT-I antigen-bearing A/PR/8/34(H1N1) for lethal infection	Tc17 and Tc1 provided equivalent survival protection against lethal infection; IFN-γ-deficient or FasL-deficient Tc17 cells were less protective, and perforin-deficient Tc1 cells were not protective	Tc17 primarily produced IL-17 and some IFN-γ, TNF-α, and IL-2; negative for granzyme B, perforin, and cytotoxicity	OT-I CD8^+^ T cells were *in vitro* polarized into Tc17 and Tc1 cells	Tc17 depends on IFN-γ and FasL; Tc1 depends on perforin for cytotoxicity and protective efficacy	(52)
IAV infection of wt and TRAIL-deficient mice; adoptive transfer of CD8^+^ T cells with/without TRAIL deficiency into infected wt mice; antibody blockage of TRAIL signaling in IAV-infected wt mice	A/PR/8/34(H1N1) for lethal infection	Greater morbidity and virus load in TRAIL-deficient vs. wt mice; transfer of TRAIL-deficient CTLs into infected mice provided less survival protection than wt CTLs; delayed virus clearance after antibody blockade of TRAIL signaling	Lower CTL cytotoxicity in TRAIL-deficient vs. wt mice	Similar magnitude of IAV-specific CTLs in wt and TRAIL-deficient mice	CTLs utilize TRAIL-mediated cytotoxicity to control IAV infection	(42, 43)

*wt, wild type*.

Both CTL effector activities (cytotoxicity and cytokine production) can contribute to protective immunity, but antigen-specific target cell destruction by CTL cytotoxicity is believed to be the primary CTL activity used for IAV clearance (11). Earlier studies showed that either perforin/granzyme- or FasL/Fas signaling-mediated apoptosis provided sufficient CTL cytotoxicity for efficient virus clearance (41). Later, TRAIL/TRAIL-DR signaling was found to contribute to CTL cytotoxicity and virus clearance (42, 43). In a non-viral infection model, in the absence of perforin, the antigen-bearing alveolar epithelial cells are not sensitive to FasL/Fas-induced cell death mediated by transferred antigen-specific CTLs, suggesting that CTLs may use different cytotoxicity mechanisms depending on the lung environmental milieu. Such differences are especially evident in studies comparing Tc1, Tc2, and Tc17 cells: after *in vitro* polarization and adoptive transfer into IAV-infected mice, Tc1 cell efficacy depended on perforin/granzymes, while Tc17s depended on FasL/Fas signaling for cytotoxicity (50, 52) and Tc1 cells did not rely on IFN-γ, but both Tc2 and Tc17 cells required IFN-γ for their protective efficacy (50–52). Thus, CD8^+^ effector T cells can protect against IAV infection via a number of redundant effector mechanisms (53). The relative contribution of each mechanism to protective efficacy may depend on a variety of factors: the differentiation status of the effector CD8^+^ T cells; the target cell type, activation status, or receptor expression; the local lung environment in the context of IAV replication; and others.

## IAV-Specific CD8^+^ T Cells Contribute to Immunopathology during IAV Infection

The two effector activities of IAV-specific CTLs allow effective specific killing of virus-infected cells but can also cause non-specific, tissue-destructive inflammation. In an earlier study, IAV infection of athymic nude mice, which cannot generate functional T cells, led to a longer survival and a slower progression of lung pathology than infection of wild-type mice, but the nude mice had a lower eventual survival rate, persistent lung injury, and higher lung virus titers (59). This study provided a first glimpse into the double-edged effects of antiviral T-cell immunity: while it provides necessary protective immunity, it does so at the cost of immunopathology. Since that study, immunopathology caused by IAV-specific CTLs has been further elucidated (Table 2).

**Table 2:** **Overview of studies demonstrating immunopathology caused by CD8^+^ T-cell responses during IAV infection**.

Experimental model	IAV subtype/infection type/pathogenicity	Disease outcome after IAV infection	Measured CD8^+^ T-cell properties		Conclusion about CD8^+^ T cells	Reference
			Frequency/number	Effector mediator		
Intranasal infection of BALB/c and athymic nude mice	Mouse-adapted A/Port Chalmers/l/73 (H3N2, MRC-9) causing lethal infection	Longer survival and slower progression of lung pathology, but eventually lower survival rate, persistent lung injury, and higher lung virus titers in nude vs. wt mice	Fewer and later lymphocytic lung infiltrates in nude mice	Nude mouse lymphocytes were non-cytotoxic and functional	T cells provided protective immunity but also contributed to immunopathology	(59)
Adoptive transfer of HA-specific CD8^+^ T cells into mice expressing an IAV HA antigen in alveolar epithelial cells	Non-viral infection model	Progressive weight loss and interstitial pneumonitis, compromised lung structure and function, inflammatory cytokine production leading to lethal lung injury in recipient mice	Adoptive transfer of HA-specific CTLs	Cytotoxicity of HA-specific CTLs, activation of target alveolar cells to produce MCP-1 cytokines	IAV-specific CTLs can cause severe lung injury after antigen recognition in the lung	(60–62)
Adoptive transfer of HA-specific CD8^+^ T cells with/without perforin deficiency into mice expressing an HA antigen in alveolar epithelial cells; antibody blockage of TNF-α signaling	Non-viral infection model	Cell death and lung injury induced by perforin-deficient HA-specific CD8^+^ T cells in recipient mice depended on TNF-α not on Fas signaling; blockage of TNF-α signaling prevented lung injury	Adoptive transfer of HA-specific CTLs	TNF-α-mediated apoptosis in bystander alveolar epithelial cells in the absence of perforin	TNF-α released by CTLs can cause severe lung injury by inducing non-specific apoptosis of alveolar epithelial cells	(63)
Adoptive transfer of HA-specific CD8^+^ T cells with/without IFN-γ deficiency into mice expressing an HA antigen in alveolar epithelial cells	Non-viral infection model	More severe lung injury after transfer of IFN-γ-producing vs. IFNF-γ-deficient HA-specific CTLs; exacerbated lung injury in State1-deficient recipients after transfer of IFN-γ-producing HA-specific CTLs	Adoptive transfer of HA-specific CTLs	IFN-γ released by IAV-specific CTLs	IFN-γ production of IAV-specific CD8^+^ T cells contributes to lung immunopathology and subsequent IFN-γ signaling of host cells regulates its inflammatory effects	(64)
Adoptive transfer of HA-specific CD8^+^ T cells with/without TNF-α deficiency into mice expressing an HA antigen in alveolar epithelial cells	Non-viral infection model	More severe lung injury, morbidity, and mortality after transfer of TNF-α-producing vs. TNF-α-deficient IAV-specific CTLs; TNF-α/TNFR1 signaling activated target alveolar cells to express inflammatory cytokines MCP-1 and MIP-2	Adoptive transfer of HA-specific CTLs	TNF-α released by IAV-specific cells and TNF-α signaling-induced inflammatory cytokines	Soluble TNF-α released by IAV-specific CD8^+^ T cells and TNF-α signaling induce inflammatory cytokine production and contribute to immunopathology	(65–67)
Adoptive transfer of IAV-specific CD8^+^ T cells into (1) wt mice or mice expressing a TNF-α signaling inhibitor, adenovirus-14.7K protein, and (2) mice expressing an IAV antigen ± expression of inhibitor	(1) A/PR/8/34(H1N1) infection and (2) non-viral infection model	(1) Less reduction of lung oxygen transfer in mice expressing TNF-α inhibitor than in wt mice, but delayed virus clearance and (2) less weight loss and lung injury in recipient mice expressing IAV antigen with TNF-α inhibitor	Adoptive transfer of IAV-specific CTLs	TNF-α released by IAV-specific CTLs	TNF-α released by CTLs and TNF-α signaling-mediated inflammation facilitate virus clearance but also cause lung injury and compromise lung function, contributing to immunopathology	(68)
Adoptive transfer of IAV-specific CD8^+^ T cells into wt or TNF-R2-deficient IAV-infected mice	A/Japan/57(H2N2) for lethal infection	Lethal infection in wt mice receiving no cells; complete survival protection in wt and TNFR2 mice receiving cells, but significant weight loss only in wt mice; no difference in virus control in mice receiving cells	Adoptive transfer of IAV-specific CTLs	TNF-α released by IAV-specific CTLs	IAV-specific CD8^+^ T cells protect mice from lethal infection but TNFR2 signaling of host cells mediates inflammation and contributes to immunopathology	(69)

One study used a non-viral infection model with CD8^+^ T CTLs specific for an IAV hemagglutinin (HA) antigen adoptively transferred into mice whose alveolar epithelial cells constitutively expressed the HA antigen. The CTL-mediated immune response is initiated when the transferred CTLs recognize the HA antigen (60). In this model, the contribution of the IAV-specific CTLs to lung immunopathology can be isolated from inflammation caused by virus replication, allowing detailed dissection of lung immunopathology caused by CTL cytotoxicity, TNF-α/IFN-γ release, and their subsequent effects on alveolar epithelial cells (Table 2). This non-viral infection model was able to cause lethal lung injury. The recipient mice showed progressive weight loss, interstitial pneumonitis, compromised lung structure and function, and increased inflammatory cytokine levels in the lung, demonstrating that IAV-specific CTLs can cause immunopathology (60, 61). Subsequent studies using this model revealed that the transferred CTLs were present only transiently (24–48 h) in the lung, and the targeted antigen-specific alveolar cells were stimulated by interaction with the CTLs to produce MCP-1 and other inflammatory mediators before they underwent apoptosis, causing lung inflammation (62). The soluble TNF-α released by the CTLs induced further inflammatory cytokine production (65–67, 69) and non-specific apoptosis of bystander alveolar epithelial cells (63), contributing to both lung injury and inflammation. Additionally, IFN-γ production by the CTLs and subsequent IFN-γ signaling contributed to lung immunopathology (64).

By isolating IAV-specific CTL responses, the non-viral infection model provided valuable insights into the CTLs’ contributions to lung immunopathology. It is also important to note that the sustained presence of IAV antigen in this model is not commonly found in the IAV infection model where the virus is eventually cleared; thus, the immunopathogenic effects of IAV-specific CTL responses were likely excessively amplified in this model and did not capture the protective effects that these cells provide in a natural infection. For example, a study using mice that express a TNF-α inhibitor in their alveolar epithelium demonstrates that TNF-α produced by the CTLs is a double-edged sword: it indeed facilitates virus clearance but at the same time contributes to severe lung immunopathology (68). Thus, it is this delicate balance between immune protection and pathology mediated by CTLs that determines the overall outcome in the host.

## Balancing the Immune Protection and Immune Pathology of CD8^+^ T Cells during IAV Infection

After IAV infection, infiltration of the lung by IAV-specific CTLs and their subsequent effector activities are essential for virus clearance but also contribute substantially to lung immunopathology; therefore, IAV-specific CTL responses must be balanced to mount effective antiviral immunity but limit tissue immunopathology. Studies using mouse models have provided insights into the complex, exquisite, control, and regulation of the magnitude of IAV-specific CTLs and their effector activities. These regulatory mechanisms include various costimulatory/inhibitory signals, cytokine/chemokine signals, and different cell–cell interactions, among others, to balance the protective (Table 3) against pathological (Table 4) effects by IAV-specific CTLs (Figure 1).

**Table 3:** **Tight regulation of CD8^+^ T-cell responses provides immune protection against IAV infection**.

Experimental model	IAV subtype/infection type/pathogenicity	Disease outcome after IAV infection	Measured CD8^+^ T-cell properties		Conclusion about CD8^+^ T cells	Reference
			Frequency/number	Effector mediator		
Wt and CTLA4Ig transgenic mice in which CD28 signaling on CD8^+^ T cells is blocked were infected with IAVs in the absence of CD4^+^ T cells	Mem/71(H3N1) infection	Virus clearance was significantly lower in CTLA4Ig transgenic than wt mice	Diminished proliferating IAV-specific CD8^+^ T cells in transgenic mice	Not described	CD28 costimulation is crucial to mount efficient IAV-specific CTLs for efficient virus clearance	(70)
IAV infection of wt, CD40L-, CD4-, or CD8-deficient mice treated with a NP-CD40L fusion protein (rAD-SNP40L)	A/PR/8/34(H1N1), lethal infection	rAD-SNP40L treatment significantly protected wt and CD40L- or CD4-deficient mice but not CD8-deficient mice from otherwise lethal infection, with reduced lung virus titer	Increased NP-specific CTLs and polyfunctional CD8^+^ T cells, along with increased NP-specific antibodies responses after treatment	NP-specific CTLs producing multiple cytokines	CD8^+^ T cells are crucial for rAD-SNP40L-mediated protection against infection	(71)
IAV infection of wt and IL-7R^a449F^-mutant mice	A/PR/8/34(H1N1) infection	More severe weight loss and failure to control lung virus titer in mutant mice vs. wt mice	Significant reduction of IAV-specific CD8^+^ and CD4^+^ T responses after IAV infection in mutant mice	Not described	IL-7 signaling is necessary for robust IAV-specific T-cell response needed for efficient virus clearance	(72)
IAV infection of wt and IL-12- or IL-18-deficient mice	X31(H3N2) infection	Delayed virus clearance from lung in IL-18-deficient mice vs. wt mice	Normal magnitudes of IAV-specific CTL responses in all groups after infection	Significantly reduced production of IFN-γ, TNF-α, and IL-2 by IAV-specific CTLs in the IL-18-deficient mice vs. wt mice	IL-18 induces optimal cytokine production by IAV-specific CTLs, which is necessary for the efficient virus clearance	(73)
Intranasal antibody blockade of PD-L1 signaling during secondary IAV infection	PR/8 challenge in X31-primed mice	Reduced weight loss and virus load in anti-PD-L1-treated mice vs. untreated mice	Increased number of IAV-specific CD8^+^ T cell in the lung in treated mice	Increased levels of granzyme B and IFN-γ production in the airway	Local PD-L1 blockade in airways enhances IAV CTL immunity, promoting virus clearance and recovery	(74)
Intraperitoneal antibody blockade of PD-L1 signaling after infection with low- and high-pathogenic IAVs	X31 and PR/8 as low- and high-pathogenic virus for sublethal and lethal infections, respectively	Anti-PD-L1 treatment reduced lung virus titer only at late infection stage and only in PR/8 infection; no effect on illness or survival	Antibody treatment significantly increased IAV-specific CD8^+^ T cells only in PR/8-infected mice	No effects on CD8^+^ T-cell functionality	Systematic PD-L1 blockade may enhance CTL protection in high-pathogenic IAV infection	(75)
IAV infection of wt and Gal-9-deficient mice; blockade of Tim-3 signaling via a Tim-3 fusion protein (Tim-3Ig) after IAV infection	X31(H3N2) infection	Gal-9-deficient mice had faster virus clearance vs. wt mice; Tim-3 blockade in wt mice resulted in increased virus control	Enhanced IAV-specific CD8^+^, CD4 T cell and antibody responses in Gal9-deficient mice; Tim-3 blockade increased IAV-specific CTL number in the airway	Not described	Gal-9/Tim-3 signaling constrains protective IAV-specific CTL immunity	(76)
IAV infection in wt and 4-1BBL-deficient mice	X31 for sublethal infection; PR/8 or PR/8-OVA for lethal infection	4-1BBL deficiency had no effect on disease severity after X31 infection, but resulted lower survival, higher virus titer, and reduced lung function after PR/8 infection vs. wt mice	Reduced IAV-specific CTLs in the lung of 4-1BBL-deficient mice only after PR/8 infection	Not described	4-1BB stimulatory signaling is induced to control IAV-specific CTL magnitude to be commensurate with IAV infection severity	(77)

**Table 4:** **Dysregulation of CD8^+^ T-cell responses contributes to immunopathology during IAV infection**.

Experimental model	IAV subtype/infection type/pathogenicity	Disease outcome after IAV infection	Measured CD8^+^ T-cell properties		Conclusion about CD8^+^ T cells	Reference
			Frequency/number	Effector mediator		
Administration of OX40 fusion proteins to IAV-infected mice	X31(H3N2) infection	Less weight loss, lower illness score, and no change in virus clearance in treated mice vs. untreated mice	Reduced total number of CD4^+^ and CD8^+^ T cells and reduced number of IAV-specific CD8^+^ T cells in treated mice	Not described	Exuberant T-cell infiltration during IAV infection contributes to immunopathology	(78)
IAV infection of wt and PD-1-deficient mice	X31(H3N2) infection	Delayed weight loss during early infection but slower recovery in PD-1-deficient mice vs. wt mice; no difference in lung function	Significantly increased number of IAV-specific CTLs in PD-1-deficient mice	Increased granzyme B and CD107a (degranulation) levels in IAV-specific, PD-1-deficient CTLs	PD-1-negative regulation of IAV CTL immunity may limit immunopathology and facilitate recovery	(79)
IAV infection of wt and Qa-1b-deficient mice; antibody blockage of NKG2A signaling	A/Japan/57(H2N2) infection	Greater pulmonary pathology in the deficient mice vs. wt mice	Not described	Enhanced TNF-α production by IAV-specific CTLs	Excessive TNF-α production by CTLs causes immunopathology	(80)
IAV infection of wt and NKG2A-deficient mice; adoptive transfer of CD8^+^ T cells with/without NKG2A deficiency to infected wt mice	A/PR/8/34(H1N1) infection	Enhanced lung injury in the NKG2A-deficient mice vs. wt mice; greater inflammation and alveolar hemorrhage after transfer of NKG2A-deficient CD8^+^ T cells	Slightly increased frequency of NP-specific CTLs in NKG2A-deficient mice	Increased production of TNF-α, IFN-γ, and IL-2 by IAV-specific CTLs in NKG2A-deficient mice	Excessive inflammatory cytokine production by CTLs causes immunopathology	(81)
IAV infection of wt and inmemTNFD1–9(K11E KI) mice (in which membrane-bound TNF-α cannot be cleaved into soluble form) and TNFR1-deficient mice; depletion of CD8^+^ T cells in TNF-α-deficient mice	Non-lethal A/PR/8/34(H1N1) infection	Greater weight loss, lung injury, and lung function compromise in both mutant mice vs. wt mice; depletion of CD8^+^ T cells in TNF-α-deficient mice attenuated lung injury	Increased number of IAV-specific CTLs and of total CD4^+^ T cells in both mutant mice vs. wt mice	TNF-α signaling via TNFR1 on CD8^+^ T cells	TNFR1 signaling on CD8^+^ T cells limits its response magnitude and the reduce CTL-mediated lung injury	(66)
IAV infection of wt and TRAIL-deficient mice	A/PR/8/34(H1N1) infection	Greater morbidity, mortality, and pulmonary pathology but no change in virus clearance in TRAIL-deficient mice vs. wt mice	Increased number of IAV-specific CTLs in the lungs of TRAIL-deficient mice due to less apoptosis and greater proliferation	Not described	TRAIL constrains excessive magnitude of IAV CTL response to prevent immunopathology	(82)

In addition to antigen stimulation, effective CD8^+^ T-cell responses are commonly believed to require secondary costimulatory signaling from DCs and/or proliferation signals from CD4^+^ helper T cells. However, during IAV infection, the primary IAV-specific CTL response is largely independent of CD4^+^ helper T cells (24). Thus, various costimulatory signals from DCs and other innate immune cells are particularly important in generating effective IAV-specific CTL immunity. Studies using specific receptor-deficient mice and/or signaling blockade have shown that both CD80/CD28 and CD70/27 costimulatory signaling are pivotal for initial activation and expansion of IAV-specific naive CD8^+^ T cells in LNs (70, 83), and CD70/27 is especially critical for accumulation of the CTLs in the infected lung by sustaining their survival (46, 84). Other costimulatory signals, including 4-1BBL/4-1BB, CD40L/CD40, and OX40L/OX40, collectively contribute to generate the optimal CTL response magnitude at late stages of infection and/or the optimal size and responsiveness of the memory CTL pool (83, 85, 86).

CD40L on the activated DC subsets provide important costimulatory signals in optimizing the magnitude of IAV-specific CTL responses in CD4^+^ T cell-independent immunity (71, 87). One study used an adenovirus-produced recombinant CD40L fused with IAV NP antigen (rAD-SNP40L) to target CD40 during IAV infection; this treatment successfully enhanced NP-specific CTL and antibody responses, reduced lung virus titers, and protected the mice from otherwise lethal IAV infection (71). Notably, type I IFN and IL-1 innate cytokines induced by IAV replication can activate DCs to increase the expression of these costimulatory molecules and thus promote IAV-specific CD8^+^ T-cell immunity (88, 89).

Another costimulatory receptor, 4-1BB, is differentially expressed on IAV-specific CTLs in lungs after infection with a low- and a high-pathogenic IAV. The higher level of 4-1BB is required to mount a higher magnitude of IAV-specific CTL responses for effective clearance of the high pathogenic IAVs (77). However, intranasal delivery of adenoviral-4-1BBL into the 4-1BBL-deficient mice after the high pathogenic IAV infection marginally improves survival at a low dose but exacerbates disease at a high dose; delivery of both doses of 4-1BBL-AdV into wild-type mice led to an increased mortality (77). This finding demonstrates an example that inducible costimulatory molecules have to be balanced to develop antiviral CTL immunity to a level commensurate with the pathogenicity of the IAVs.

Costimulatory signals can also result in exuberant T-cell inflammation that contributes to immunopathology. OX40 is a costimulatory receptor expressed only on activated T cells, and its interaction with OX40L on DCs imparts a survival signal to the T cells, preventing activation-induced cell death (90). Blockage of OX40L/OX40 signaling during IAV infection by administration of an OX40 fusion protein significantly reduced the magnitude of both IAV-specific CTL and CD4^+^ T-cell responses (78). However, unlike the effects of other costimulatory signals described above, reduction of T-cell responses by OX40L/OX40 blockade ameliorated disease symptoms without compromising effective virus clearance (78). Clearly, OX40L/OX40 signaling during IAV infection is not necessary for virus clearance but contributes to immunopathology.

On the other hand, there are also various coinhibitory receptors on T cells, mediating inhibitory signals to activated T cells. Coinhibitory signaling serves to prevent tissue damage by limiting the response magnitude but also may constrain the effective immunity necessary for protective efficacy. For example, tissue-expressed Galectin-9 (Gal-9) binds to its receptor Tim-3 on T cells to limit the response magnitude. Gal-9 deficiency or blockade of Tim-3 signaling resulted in more robust virus-specific CTL and antibody responses to IAV infection, leading to a more rapid virus clearance and recovery (76), suggesting that Gal-9/Tim-3 signaling constrains effective antiviral immunity. NKG2A/CD94, another inhibitory receptor originally described mainly on NK cells, was found to be expressed on IAV-specific CTLs during IAV infection. Deficiency of NKG2A or its ligand Qa-1b, or blockage of Qa-1b/NKG2A signaling, resulted in a greater pulmonary pathology accompanied by an enhanced TNF-α production by the IAV-specific CTL cells (80, 81); therefore, NKG2A-mediated negative signaling truly limits CTL immunity-mediated lung injury. Another inhibitory receptor, CD160, was also recently found on CD8^+^ T cells during influenza infection, and CD160-ligand/CD160 signaling reduced the proliferation capacity and perforin expression of the CD8^+^ T cells (91), although the role of this negative signaling in the outcome of the IAV infection is unclear.

PD-1 is another inhibitory receptor found to be unregulated on IAV-specific CTLs after primary or secondary IAV infection, and its ligand PD-L1 was found to be expressed on lung epithelial cells and innate immune cells (74, 75, 79). PD-L1/PD-1 signaling leads to severe functional impairment of CTLs. Inhibition of PD-L1/PD-1 signaling during IAV infection by blocking either PD-1 or epithelial PD-L1 with antibodies in wild-type mice enhanced CTL function and subsequent virus clearance and ameliorated disease severity (74, 75). However, in PD-1-deficient mice, CTL magnitude and function were improved after IAV infection, but recovery was delayed (79), suggesting that PD-1-negative signaling is required to limit the adverse effects of the IAV-specific CTL responses. Clearly, this negative signaling is necessary to balance CTLs’ role in virus control, speed of recovery, and immunopathology. Interestingly, another study found that only infection with highly pathogenic but not with low pathogenic IAV induced a greater PD-1 expression on the IAV-specific CTLs (75), demonstrating that the optimal magnitude of antiviral CTL immunity can be dampened as a consequence of the high pathogenicity of a particular IAV.

Plasmacytoid DCs are also found to negatively regulate the magnitude of CTL responses in LNs after lethal infection with a high dose of IAV or with a highly pathogenic H5N1 IAV but not after sublethal infection (17, 18). After the lethal infections, pDCs accumulating in the LNs showed a high expression of FasL, driving the elimination of Fas^+^ CD8^+^ cells. The dampened IAV-specific CTL responses enhanced the lethality of the infections, providing another example of how the optimal magnitude of antiviral CTL responses can be compromised in infections with IAVs of high pathogenicity, consistent with the clinical findings of lymphopenia in patients infected with highly pathogenic H5N1 IAVs (92).

Various soluble cytokines induced during IAV infection also play an important role in the trafficking, survival, and effector activity of CD8^+^ effector T cells required for optimal antiviral CTL responses. For example, IL-15 is chemotactic, responsible for the migration of IAV-specific CTLs to the infected lung (93) and also contributes to the survival of the CTLs in the infected lung (27, 93). Both IL-7 (72) and IL-18 (73) are necessary for the development of a robust IAV-specific CTL response required for efficient virus clearance. IL-12 was also shown to be important for CTL cytotoxicity *in vitro* (94).

Two signaling molecules, TNF-α and TRAIL, used in the effector mechanisms of CTLs, can also constrain the magnitude of IAV-specific CTL responses to prevent the excessive damage. TRAIL deficiency (82) and TNF-α deficiency (66) increased the number of IAV-specific CTLs accompanied with decreased apoptosis and increased proliferation but increased host morbidity and mortality after IAV infection. Thus, both TRAIL/TRAIL-DR signaling and TNF-α/TNFR1 signaling have a dual role during IAV infection depending on the cell type in which the receptors are expressed: they can kill IAV-infected cells for immune protection and also negatively regulate the magnitude of the CTL response to reduce the likelihood of immunopathology.

Various types of innate and adaptive immune cells, including IAV-specific CTLs, infiltrate the IAV-infected lung. These infiltrating cells, the lung-resident cells, and the cytokines that they produce compose the local inflammation milieu, which can further shape the antiviral CTL response. For example, maintaining an optimal magnitude of protective IAV-specific CTL responses requires interactions with multiple non-migratory DC subsets in the infected lung for essential survival and proliferation signals (26–28). An inflammatory monocyte population (sometimes referred to as TipDCs) is recruited from bone marrow to the infected lung by CCL2/CCR2-mediated chemoattraction. TipDCs and the TNF-α that they produce promote IAV-specific CTL expansion and survival in the lung, which are required for optimal protection (95). The IAV-infected lung is also characterized by a large neutrophil infiltration. Migrating neutrophils leaves behind long-lasting CXCL12-enriched trails and routes into the infected lung to facilitate efficient CD8^+^ T-cell migration and localization to the infection site, which is required for optimal CTL infiltration and effective virus clearance (96). In the lung interstitium, neutrophils also serve as antigen-presenting cells that promote IFN-γ production by CTLs (97). NKT cells are recruited to the lung, where they themselves have a protective role against IAV infection (98). Furthermore, NKT cells also promote the accumulation of CD103^+^ DCs in the LNs and promote the subsequent IAV-specific CD8^+^ T-cell response (99). Finally, IAV antigen-specific Treg cells are found in both primary and secondary IAV infections, negatively regulate the proliferation of IAV-specific CTLs, and limit the pulmonary inflammation during secondary IAV infection (100).

During IAV infection, not only is the magnitude of the antiviral CTL response regulated by multiple signals from various cells, but also the antiviral effector activities of the CTLs are dictated by the target cell type encountered and the costimulatory signals and antigen intensity the target cell carries. For example, CD45^+^ inflammatory mononuclear cells in the lung interstitium, such as CD11c-high cells, which express costimulatory ligands (CD80/86), stimulate both CTL cytotoxicity and release of inflammatory cytokines, but CD45^−^ respiratory epithelial cells expressing few stimulatory ligands trigger only CTL cytotoxicity (14, 101). Consistent with this finding, after adoptive transfer into IAV-infected mice, highly cytotoxic Tc1 cells were found to localize near the infected airway epithelium, but Tc2 cells localized within the clusters of other inflammatory cells distant from epithelium (50), suggesting that different subsets of CD8^+^ T cells might interact with the subsets of innate cells resulting in diverse effector activities.

During the CTL and target cell interaction, CTL TCR recognition of its cognate pMHCI complex on the target cells determines the specificity of CTLs, while the strength of TCR/pMHCI recognition fine-tunes the extent of CTL effector activities (102–104). The requirements of TCR occupancy and signal strength to activate CTL cytotoxicity are very low to minimal, allowing rapid and specific target cell killing, while the requirement for the induction of inflammatory cytokines is much higher, limiting the extent of non-specific inflammation induced by the cytokines (102–104). Thus, the division of specialized roles among the different antigen-presenting cells and the two activation thresholds for the two CTL effector activities enable specific target cell killing while not necessarily causing excessive inflammation in the infected lung. In addition, from a temporal view of IAV replication and the antiviral CTL response, it is also reasonable to speculate that higher viral antigen load and stronger TCR signals on CTLs at the early phase of infection may promote terminal CTL effector activity, while the lower antigen load and TCR signals on CTLs at late phases of infection or after virus clearance may promote CTL memory potential or differentiation.

In summary, during IAV infection, both the magnitude and effector activities of the CTL response are exquisitely regulated by various stimulatory and inhibitory signals, cytokines, and chemokines from a variety of cell types to achieve the goal of immune protection while minimizing potential immunopathology.

## CD8^+^ T-Cell Response is a Positive Correlate of Protective Immunity Against Heterosubtypic IAV Infection

Human populations are challenged constantly by mutated variants of circulating seasonal IAVs (currently H1N1 and H3N2) and occasionally by novel pandemic strains. An estimated 5–20% of the population worldwide is infected annually with a seasonal IAV (105). Thus, human IAV infections are almost invariably at least secondary, except in young children. Therefore, it is of great importance to understand heterosubtypic IAV immunity, which is generated by a given IAV subtype but offers some protection against challenge with another IAV subtype. In the face of a heterosubtypic IAV infection, the neutralizing antibodies generated by IAV-specific B cells can recognize only the specific IAV surface proteins from a previous infection, while IAV-specific CTLs target viral peptides that usually are derived from IAV internal proteins and are relatively conserved across different subtypes (106). Thus, cross-reactive IAV-specific CTLs generated by previous IAV infection are an essential component of heterosubtypic immunity (107, 108), as shown consistently in both animal and human studies.

Animal studies (Table 5) using a prime/challenge model with IAVs of different subtypes have shown that the cross-reactive CD8^+^ T cells generated by a first IAV infection are unable to prevent secondary IAV infection but clearly ameliorate morbidity and mortality, reduce the virus load, and accelerate recovery; this protection can even boost survival after an otherwise lethal challenge with H5N1 or H7N9 IAV (109–115). These studies have consistently demonstrated that CD8^+^ T cells mediate heterosubtypic immunity. One study found that the best predictor of protective efficacy against secondary infection was the overall size of the memory CTL pool generated by the priming infection, rather than conservation of known CD8^+^ epitopes between viruses (114). The size of the memory CTL pool may serve as a proxy indicator of the robustness and quality of the primary immune response and may be associated with the activation status and quality of memory CTLs. Meanwhile, this finding also illustrates our incomplete understanding of the full measure of CD8^+^ epitope diversity and other heterologous immune mechanisms generated by initial priming, which warrants future studies.

**Table 5:** **Overview of animal studies showing that IAV-specific CD8^+^ T-cell responses are a correlate of protection against heterosubtypic secondary IAV infection**.

Experimental model		Disease outcome after second IAV infection	Measured CD8^+^ T-cell properties		Conclusion about CD8^+^ T cells	Reference
First IAV infection (priming)	Second IAV infection (challenge)		Frequency/number in second responses	CTL cross-reactivity		
Udorn(H3N2) by IV, IP, or IN routes	4 weeks later, A/PR/8/34(H1N1) lethal infection	20% survival in primed mice	Increased heterosubtypic CTL effectors in primed mice	Heterosubtypic CTL cytotoxicity detected during memory and second responses	CTLs are associated with heterosubtypic immunity	(109)
A/Quail/HK/G1/97(H9N2) by IN route	4 weeks later, A/HK/156/97(H5N1) lethal infection	Complete survival with less weight loss in primed mice	Not described	Heterosubtypic CTL cytotoxicity detected during memory response	CTLs are associated with heterosubtypic protection	(110)
X31(H3N2) by IN route	4 weeks later, A/PR/8/34(H1N1) lethal infection	Accelerated virus clearance, reduced clinical signs, reduced lung lesions, and increased survival rate in primed mice	Significantly greater NP-specific CTL population in primed mice	Not described	Enhanced cross-reactive NP-specific CTL response is associated with protection	(111)
A/HongKong/2/68(H3N2) or a respiratory syncytial virus by IN route	4 weeks later, A/Indonesia/5/05 (H5N1) lethal infection	Reduced clinical signs, weight loss, mortality, and lung virus replication in IAV-primed mice but not in RSV-primed mice	Greater expansion of cross-reactive NP-specific CTLs in primed mice	Cross-reactive NP-specific CTLs	Expanded cross-reactive NP-specific CTLs are associated with protection	(112)
A/Kawasaki/173/01(H1N1) by a combination of routes (nasal, ocular, and tracheal) in rhesus macaques	4 months later, A/California/04/09(pandemic H1N1) infection	Faster virus clearance in primed animals	Earlier detection and higher number of activated CD8^+^ T cells in blood and lung of primed animals	Cross-reactive IAV-specific IFN-γ producing T cells	Cross-reactive CD8^+^ T cells is involved in protection	(113)
A/Chicken/Hong Kong/TP38/03(H9N2), A/Hong Kong/33982/09 (H9N2), A/California/4/09(H1N1), or A/PR/8/34(H1N1) by IN route	10 weeks later, A/Anhui/01/13(H7N9) lethal infection	Lower morbidity and mortality, pulmonary virus load, and time to clearance in primed mice	Earlier and greater airway infiltration by IAV-specific CTLs in primed mice	IAV-specific CTLs targeting conserved or cross-reactive epitopes were detected during memory and second responses	Cross-reactive IAV-specific CTLs contribute to heterosubtypic protection and the magnitude of the IAV-specific CTL memory pool are the best predictors of protective efficacy	(114)
A/PR/8/34(H1N1) by IN route	5 weeks later, A/Anhui/01/13(H7N9) lethal infection	Lower lung virus titer, morbidity, and mortality in primed animals	Not described	Cross-reactive IAV-specific CD4^+^ and CD8^+^ cells detected during memory response	Cross-reactive T cells are associated with protection	(115)

*IV, intravenous; IP, intraperitoneal; IN, intranasal*.

Due to the many challenges posed by human studies, the role of CD8^+^ T cells in mediating heterosubtypic protection against illness caused by natural IAV infection is far from certain, but studies using human samples have provided some useful insights. A number of studies of human peripheral blood cells have demonstrated the existence of cross-reactive CD8^+^ T-cell immunity between distinct heterosubtypic IAVs, such as between seasonal IAVs and 2009 pandemic H1N1 IAV (116, 117), between the 1918 pandemic H1N1 and 2009 pandemic H1N1 IAVs (118), between seasonal IAVs and avian H5N1 IAVs (119), and between seasonal IAVs and the novel H7N9 IAVs (115, 120, 121). These studies consistently show that prior IAV infection in humans can generate a measure of cross-reactive, or “heterosubtypic,” CD8^+^ T-cell-mediated immunity against other serologically distinct IAVs, for potential immune recall responses upon another IAV infection.

A few studies in humans have demonstrated that the cross-reactive IAV-specific CD8^+^ T-cell response is a positive correlate of cross-protective immunity against secondary IAV infection (Table 6). An earlier human study using experimental H1N1 challenge showed that the extent of CTL cytotoxicity in the blood of subjects at 2 days after challenge was closely associated with less virus shedding, faster virus clearance, and less severe symptoms (122), providing the first evidence that the preexisting cross-reactive CTL response is a correlate of clinically cross-protective immunity (day 2 was too early to generate CTL responses to the challenge IAV). The 2009 H1N1 pandemic and the recent emergence of human infections with H7N9 IAV in China have provided unique opportunities to study the natural heterosubtypic IAV infection and cross-protective response in the absence of the specific neutralizing antibodies. One study followed a cohort of healthy adults through the pandemic waves in the UK (123), and another examined a cohort of hospitalized patients with severe H7N9 infection in China (124). Both studies found that the magnitude of the cross-reactive CD8^+^ T-cell response was positively correlated with a favorable clinical outcome in the patients. Although these findings are consistent with those obtained in mice, it is noteworthy that human studies often involve complex and uncontrollable variables, including demographic, environmental, and genetic differences. As a consequence, extreme care must be taken in interpreting the results of human studies.

**Table 6:** **Overview of human studies showing that cross-reactive CD8^+^ T-cell responses are a correlate of protective immunity against human IAV infection**.

Human IAV infection	Findings in CD8^+^ T cells and disease outcome after IAV infection	Conclusion about CD8^+^ T cells	Reference
Experimental infection with A/Munich/1/79(H1N1) IAV	Greater CD8^+^ T-cell cytotoxicity in patient blood samples at day 2 after inoculation was a correlate of lower virus shedding, faster virus clearance, and lower disease symptom score	High CTL responses is positively associated with recovery from seasonal H1N1 IAV infection	(122)
Natural infection with 2009 pandemic H1N1 IAV	Higher proportion of preexisting CD8^+^ T cells to conserved epitopes was observed in individuals who developed less severe illness	Preexisting CD8^+^ T cells specific for conserved IAV epitopes were a positive correlate of cross-protection against the severity of H1N1 IAV infection	(123)
Natural infection with H7N9 IAV	Patients with shorter hospitalization had an early, prominent H7N9-specific CD8^+^ T-cell response, while those with longer hospitalization had delayed or no T-cell activity	A robust CD8^+^ T-cell memory response is positively associated with protection against H7N9 IAV infection	(124)

## Concluding Remarks

Due to their naturally high mutation rate and their ability to generate genetic reassortants, IAVs pose a continuing threat to human populations. In order to develop better therapeutic and vaccination strategies to appropriately modify CTL immunity for protection against IAV infection, it is a great need to better understand the regulation and balance of the immune protection and pathology involved in virus-specific CTL immunity during IAV infection. We argue that the counterbalance of these effects depends on multiple layers of host and viral factors, including complex host mechanisms that regulate CTL quantity, quality, and effector activities; the pathogenic profile of the IAV, especially its ability to induce different innate host response milieus in which CTL immunity is primed and regulated; and the historical immune context of influenza infection (e.g., primary, secondary, and postvaccination). We propose that future research efforts should refine our understanding of key host and viral parameters, their points of interaction, and the effects of these interactions under different contexts of influenza occurrence (primary, secondary, postvaccination challenge, or heterologous infection), especially to differentiate those that constrain optimally effective CTL antiviral immunity from those truly necessary to restrain CTL-mediated non-specific immunopathology. Further translational research efforts can then focus on developing preventive or therapeutic means to modulate these parameters and interactions to obtain the best clinical outcomes.

## Author Contributions

PT assisted in review conception, planning, and editing. SD conceived, planned, and wrote the review and designed the figure.

## Conflict of Interest Statement

The authors declare that the research was conducted in the absence of any commercial or financial relationships that could be construed as a potential conflict of interest.
